# The IMPACT framework for evaluating generative AI in critical care: development and multinational consensus validation

**DOI:** 10.1016/j.aicoj.2026.100078

**Published:** 2026-05-12

**Authors:** Yu-Chang Yeh, Ming-Chieh Shih, Daniel De Backer, Leo Anthony Celi, Kay Choong See, Tomoko Fujii, Lowell Ling, Wasineenart Mongkolpun, Hsiang-Wei Hu, Hsuan-Yu Chen, Wei-Cheng Chen, Bernard Cholley, Kean Khang Fong, Ho-Geol Ryu, Sungwon Na, Moritoki Egi, Wing-Sum Chan, Kuan-Fu Chen, Rishikesan Kamaleswaran, Yu-Chen Chuang, Chi-Ju Yang, Wei-Ling Hsiao, Sheng-Ru Lai, David Ku, Ahsina Jahan, Greg S. Martin, the IMPACT Group

**Affiliations:** a Department of Anesthesiology, National Taiwan University Hospital, Taipei, Taiwan; b School of Medicine, College of Life Sciences and Medicine, National Tsing Hua University, Hsinchu, Taiwan; c Department of Intensive Care, CHIREC Hospitals, Université Libre de Bruxelles, Brussels, Belgium; d Laboratory for Computational Physiology, Massachusetts Institute of Technology, Cambridge, MA, USA, Department of Medicine, Beth Israel Deaconess Medical Center, Boston, United States of America; e Division of Respiratory and Critical Care Medicine, Department of Medicine, National University Hospital, Singapore; f Department of Intensive Care, Jikei University Hospital, Tokyo, Japan; g Department of Anaesthesia and Intensive Care, The Chinese University of Hong Kong, Hong Kong SAR, China; h Division of Critical Care Medicine, Department of Medicine, Siriraj Hospital, Mahidol University, Bangkok, Thailand; i Taiwan Artificial Intelligence Association, Taipei, Taiwan; j Department of Orthopedic Surgery, National Taiwan University Hospital, Taipei, Taiwan; k Respiratory Intensive Care Unit, China Medical University Hospital, Taichung, Taiwan; l Department of Anesthesiology and Intensive Care Medicine, Hôpital Européen Georges-Pompidou, AP-HP, Paris, France; m Department of Medicine, Queen Elizabeth Hospital, Kota Kinabalu, Malaysia; n Department of Anesthesiology and Pain Medicine, Seoul National University Hospital, Seoul, Republic of Korea; o Department of Anesthesiology and Pain Medicine, Severance Hospital, Seoul, Republic of Korea; p Department of Anesthesiology and Intensive Care Medicine, Kyoto University Hospital, Kyoto, Japan; q Department of Anesthesiology, Far Eastern Memorial Hospital, New Taipei, Taiwan; r Clinical Informatics and Medical Statistics Research Center, Chang Gung University, Taoyuan, Taiwan; s Department of Surgery and Department of Anesthesiology, School of Medicine, Duke University, Durham, NC, United States of America; t Information Technology Office, National Taiwan University Hospital, Taipei, Taiwan; u Department of Pharmacy, National Taiwan University Hospital, Taipei, Taiwan; v Department of Nursing, National Taiwan University Hospital, Taipei, Taiwan; w Department of Dietetics, National Taiwan University Hospital, Taipei, Taiwan; x Monash Health, Melbourne, Australia; y Department of ICU and Emergency, Brahmanbaria Medical College Hospital, Brahmanbaria, Bangladesh; z Division of Pulmonary, Allergy, Critical Care and Sleep Medicine, School of Medicine, Emory University, Atlanta, United States of America

**Keywords:** Generative artificial intelligence, Critical care, Clinical decision support, Content validity, Consensus

## Abstract

**Background:**

Generative artificial intelligence (GenAI) is increasingly used for clinical decision support in critical care, yet standardized methods for evaluating GenAI content in intensive care settings are lacking. Existing metrics assess textual similarity but fail to capture clinical accuracy, reasoning quality, or urgency.

**Methods:**

We developed and validated the IMPACT framework through a five-phase multinational panel consensus process. Reporting adhered to the ACCORD guideline. A steering committee of eight persons provided clinical and methodological oversight. Panelists were recruited through purposive sampling to ensure geographic and multidisciplinary representation. Content validity was assessed using the Content Validity Ratio (CVR) and Item-level Content Validity Index (I-CVI), with retention thresholds set at 70% agreement and I-CVI ≥0.80.

**Results:**

A total of 58 panelists from 12 countries and regions participated, with 42 completing formal consensus voting. Participants included intensivists, physicians with AI research expertise, information technology specialists, and other critical care professionals. All six IMPACT domains exceeded validity thresholds (mean agreement 89.3%, CVR = 0.79, I-CVI = 0.92). Of 24 candidate subitems, 21 met retention criteria (mean agreement 85.7%, CVR = 0.71, I-CVI = 0.90). Three subitems were removed due to insufficient consensus and conceptual overlap. The validated framework comprises six domains with 21 subitems.

**Conclusions:**

The IMPACT framework provides a consensus-validated approach for evaluating GenAI clinical decision support in intensive care, addressing gaps in current evaluation methods.

## Graphical abstract

**Figure:**
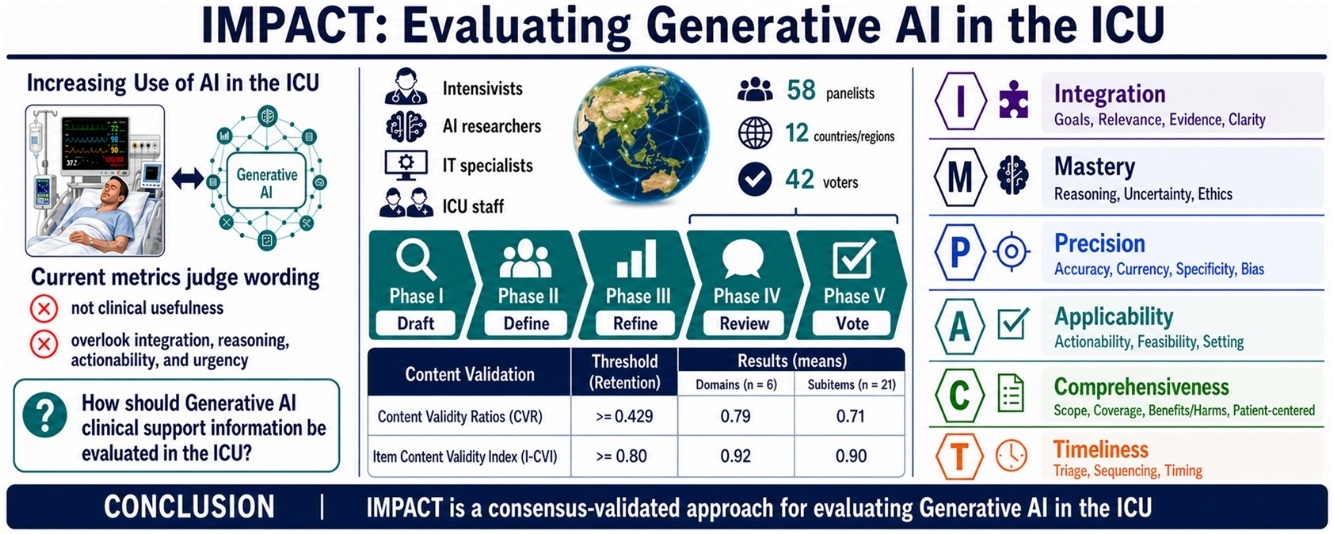

## Introduction

Generative artificial intelligence (GenAI) is transforming clinical decision support in critical care [[1], [2], [3]]. In intensive care units (ICUs), clinical decisions are complex and carry significant risk. Diagnostic errors affect up to 40% of ICU patients, and major errors double mortality risk [4,5]. GenAI refers to a broad class of AI systems that produce new content such as text, images, audio, code, or structured outputs by learning patterns from large training datasets. Large language models (LLMs) are a specific subset of GenAI focused on natural language understanding and generation, while the broader GenAI field also includes image, audio, and multimodal generative models that extend beyond text. In simulated settings, large language models (LLMs) have demonstrated improved diagnostic reasoning [6,7].

However, a gap exists between simulated and real-world performance. LLMs perform worse than clinicians on actual clinical cases [8]. They struggle with clinical guidelines and show problematic sensitivity to information ordering [8]. LLMs achieve 84–90% accuracy on United States Medical Licensing Examination (USMLE) exams but only 45–69% on practice-based assessments [9,10]. Medical licensing exam benchmarks are fundamentally limited as proxies for clinical competence [11], and LLMs perform markedly lower on clinical reasoning assessments than on traditional knowledge-based benchmarks [12]. Top-scoring models often fail on real patient records [9,12]. A major reason is inadequate evaluation metrics. Common metrics like BLEU, ROUGE, and BERTScore assess textual similarity but miss clinical accuracy and reasoning quality [[13], [14], [15]].

Existing tools like DISCERN provide useful foundations but are insufficient for intensive care settings [16]. Recent guidelines (TRIPOD + AI, TRIPOD-LLM, DECIDE-AI, CHART) offer valuable reporting frameworks [[17], [18], [19], [20]]. However, no unified instrument addresses the specific challenges of evaluating GenAI in critical care. Outputs must demonstrate sound reasoning, be actionable, fit the clinical context, and address urgency appropriately.

To address this gap, we developed the IMPACT framework, an acronym derived from its six domains: Integration, Mastery, Precision, Applicability, Comprehensiveness, and Timeliness. We used consensus methodology because no gold standard criteria exist for evaluating GenAI outputs in critical care, and empirical validation alone cannot capture the tacit clinical knowledge needed in intensive care settings. This article describes the framework development and reports content validation through multinational panel consensus involving ICU clinicians, physician scientists, and information technology (IT) specialists. Our aim is to provide a structured tool for evaluating GenAI clinical decision support in critical care.

## Methods

### Study design, objective, and oversight

This study employed a structured, multi-phase consensus methodology to validate the conceptual completeness, relevance, and clarity of the IMPACT Evaluation Framework for assessing GenAI clinical decision support content in critical care. Reporting adhered to the ACCORD (ACcurate COnsensus Reporting Document) guideline for consensus methods in biomedicine [21]. The completed ACCORD checklist is provided in Additional File 1. In the absence of a dedicated registry for panel consensus studies, we ensured methodological transparency by providing a comprehensive protocol description in the Methods. A steering committee of eight persons (including the chair, Y.C.Y., with seven members G.S.M., K.C.S., L.L., W.M., H.Y.C., W.C.C., and M.C.S.) provided clinical and methodological oversight across all phases. Committee members were invited by the chair and participated voluntarily. They approved all item wording revisions. One committee member (M.C.S.), a physician biostatistician, provided additional statistical oversight for the content validity analysis.

### Panel eligibility, selection, and composition

We used purposive sampling to maximize geographic and multidisciplinary representation. Panelists were eligible if they had leadership roles in national critical care societies, clinical practice in the ICU, physician expertise in AI research relevant to clinical applications, or professional experience in information technology and clinical informatics. The target panel size balanced breadth of representation with feasibility for iterative review and voting. The chair personally invited 60 potential panelists via email, WhatsApp, and LINE, with reminder messages sent through the same channels. Participating panelists could nominate additional panelists; all nominations were reviewed and approved by the chair and steering committee. Participation was voluntary without financial incentives or reimbursement. No patients, carers, or members of the public participated in the design, item generation, panel review, or voting, as the framework was intended to evaluate GenAI clinical decision support content for clinicians in critical care.

### Development process and item generation

**Fig. 1:**
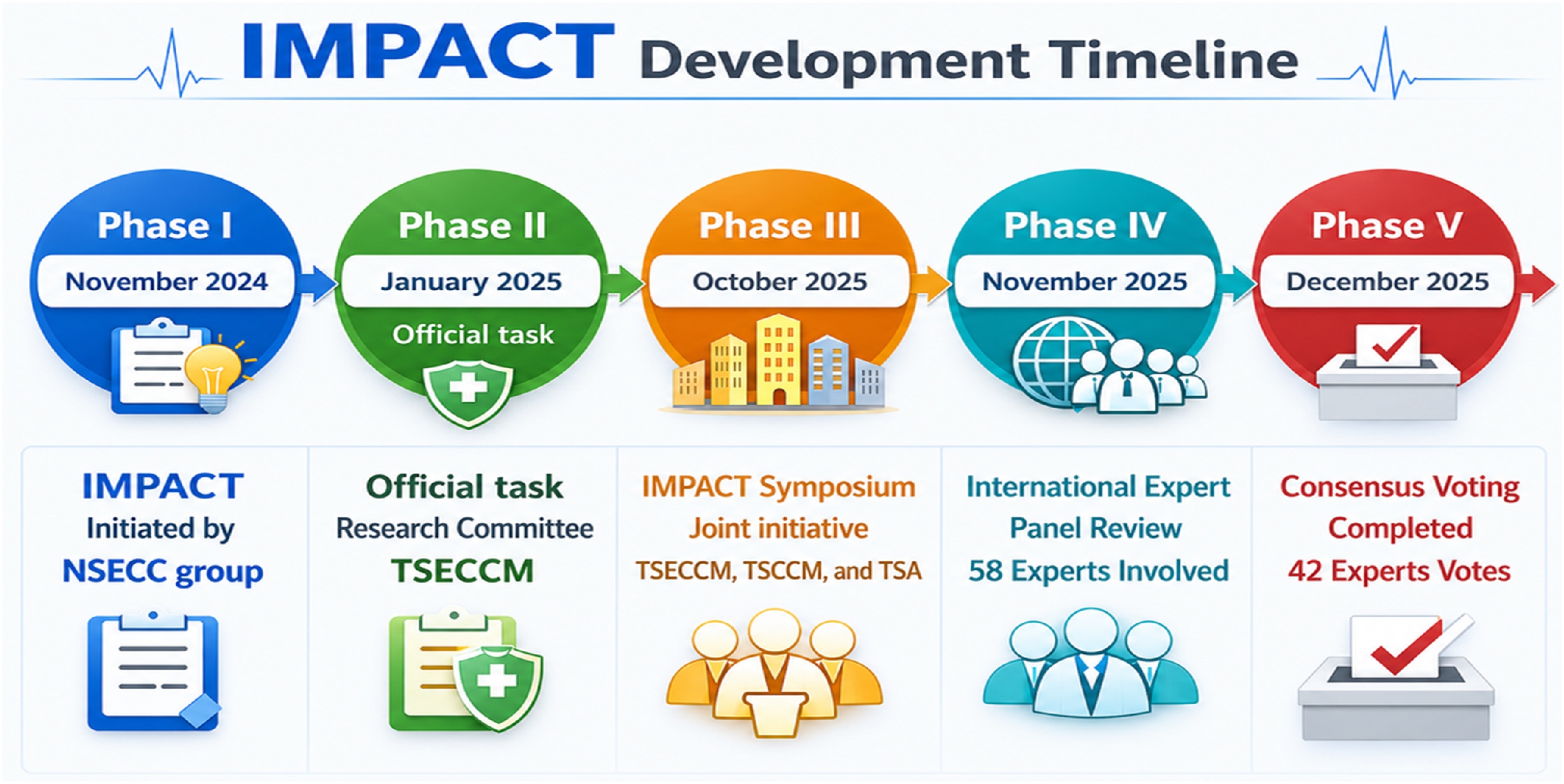
IMPACT Framework Development Timeline. The IMPACT framework was developed through a five-phase multinational panel consensus process from November 2024 to December 2025. The process progressed from initial conceptualization by the NSECC group, formal adoption by the TSCCM Research Committee, a joint symposium involving TSECCM, TSCCM, and TSA, multinational panel review with 58 panelists from 12 countries and regions, to final consensus voting completed by 42 panelists. Figure prompt by YC Yeh; image generated using GPT-5.2.

The IMPACT Evaluation Framework was developed through a five-phase consensus process (Fig. 1). Phase I (November 2024) initiated the framework concept within the National Taiwan University Hospital (NTUH) Smart Emergency and Critical Care (NSECC) group. The NSECC group conducted a focused review of established appraisal frameworks for online health information quality, and emerging evaluation approaches for AI-generated clinical content. PubMed was searched between November and December 2024 using terms including 'health information quality,' 'clinical decision support evaluation,' 'AI evaluation framework,' and 'large language model assessment.' This review identified established instruments including DISCERN [16], QUEST [22], HONcode [23], JAMA benchmarks [24], and DARTS [25]. Using these appraisal frameworks as a foundation, candidate domains and subitems were drafted to reflect the unique evaluation needs of GenAI in ICU settings. Phase II (January 2025) formalized IMPACT as an official task of the Research Committee of the Taiwan Society of Emergency and Critical Care Medicine (TSECCM). Background rationale, intended use, and working definitions were discussed during committee meetings. Phase III (October 2025) facilitated comprehensive interdisciplinary discussion through the IMPACT Symposium, a joint hybrid initiative of the Taiwan Society of Emergency and Critical Care Medicine, the Taiwan Society of Critical Care Medicine (TSCCM), and the Taiwan Society of Anesthesiologists (TSA). The symposium included lectures and group discussions. Participants included technical experts (IT professors, engineers), clinical staff (physicians, nurses, pharmacists, and dietitians), and trainees (residents, medical and IT students), with the majority from Taiwan and several from Singapore and Hong Kong. Feedback was gathered through group discussion at the hybrid symposium, which also confirmed the planned Content Validity Ratio (CVR) and Item-level Content Validity Index (I-CVI) approach. This symposium also served as a pilot evaluation of the draft framework domains, subitem definitions, and survey format; participant feedback led to refinements in item wording and presentation before the formal international panel review and voting in Phases IV and V. Phase IV (November 2025) conducted an international panel review. Panelists received a structured information package that included the draft IMPACT framework with domain definitions and subitem descriptions, a summary of the foundational appraisal instruments and their relevance to GenAI evaluation, and the rationale for the proposed CVR and I-CVI methodology. Panelists reviewed the contents independently and provided written feedback via email or messaging platforms. Phase V (December 2025) quantified content validity through structured voting. After voting, aggregated quantitative results including the number and percentage of panelists endorsing each domain and subitem were shared with all panelists for review. Panelists provided qualitative feedback on item wording and interpretation via email and messaging platforms, which informed the steering committee's final decisions on item retention and wording refinements.

### Framework structure

The final IMPACT framework comprises six domains with defined subitems: Integration (clear goals, clinical relevance, evidence-based embedding, clarity and consistency); Mastery (correct clinical reasoning, mechanistic understanding, transparency of uncertainty, adherence to ethical standards); Precision (accuracy of content, up-to-date knowledge, specificity of recommendations, bias assessment); Applicability (actionable implementation, achievable feasibility, appropriate setting, alternative options); Comprehensiveness (full scenario scope, multidomain coverage, benefits and harms, patient-centered care); and Timeliness (urgency-based triage, priority sequencing, timing and intervals, next-step planning).

### Voting procedures

Voting was conducted during two online meetings using Slido for live polling, with Google Forms available for panelists who encountered technical issues or could not attend. Meetings and survey materials were primarily in English, with a Mandarin instruction document provided for Taiwanese panelists. Each domain and subitem was presented as a separate statement with its definition and prespecified response options. A single voting round was conducted.

### Content validity assessment

To evaluate content validity of both domains and subitems, we used two complementary quantitative indices: the CVR and the I-CVI. CVR quantifies the degree of expert agreement on whether an item is essential, while I-CVI quantifies its relevance, so that the two indices together capture both necessity and relevance of the retained items. For CVR, essentiality was assessed using the Lawshe method [26]. Panelists rated each domain and subitem on a 3-point scale: 1 = necessary, 2 = useful but not necessary, and 3 = not necessary. CVR was calculated as CVR = (Ne − N/2) / (N/2), where Ne represents the number of panelists rating the item as necessary (score of 1) and N represents the total number of panelists. CVR values range from −1 to +1, and a value greater than zero indicates that more than half of the panelists judged the item to be essential, with values approaching 1.0 reflecting stronger consensus. This metric enables systematic identification and refinement of weak or potentially irrelevant items. Although the minimum critical CVR value required for a 42-member panel is 0.29 [27], we adopted a stricter predefined criterion of CVR ≥ 0.429, corresponding to at least 70% necessary ratings, to ensure high panel consensus. For I-CVI, panelists rated relevance of each domain and subitem on a 4-point scale: 1 = not relevant, 2 = somewhat relevant, 3 = quite relevant, and 4 = highly relevant [28,29]. Ratings were dichotomized, with scores of 3 or 4 indicating acceptable relevance, and I-CVI was computed as the proportion of panelists rating the item as relevant. Although the commonly accepted I-CVI threshold for acceptable content validity is 0.78, we adopted a stricter predefined criterion of I-CVI ≥ 0.80 to ensure robust content validity given the clinical safety implications of GenAI recommendations in critical care. If a panelist did not provide a rating for an item, the response was conservatively assigned as 3 (not necessary) for CVR and 1 (not relevant) for I-CVI, ensuring that only items with explicit panel endorsement achieved validity thresholds.

### Data handling, ethics, and governance

Panel characteristics were summarized using descriptive statistics. Quantitative voting results were used to calculate CVR and I-CVI for each domain and subitem. Participation was voluntary. Voting was anonymous at the panel level; panelists could not see how others voted. The chair maintained a participant roster to monitor completion and arrange follow-up voting. Only the chair could link participants to responses. Results were analyzed and reported in aggregated form. The chair and steering committee supervised comment synthesis and approved final wording revisions. Chair and steering committee members participated in Phase V voting, and their votes were included in CVR and I-CVI calculations.

## Results

### Panel composition

A total of 58 panelists participated in the international panel review. The panel comprised 34 intensivists, five non-ICU physicians with research interests in AI, five university-based information technology faculty, four information technology engineers, three ICU nurses, two ICU pharmacists, and one each of an ICU nephrologist, ICU dietitian, extra-corporeal membrane oxygenation physician, physician biostatistician, and physician ethicist. Participating panelists represented 12 countries and regions: Taiwan (n = 36), the United States (n = 4), Australia (n = 3), two each from Hong Kong, Japan, Malaysia, Singapore, South Korea, and Thailand, and one each from Bangladesh, Belgium, and France. Among the 42 voting panelists, 25 were from Taiwan and 17 were from 11 other countries and regions. The geographic and professional breakdown of panelist participation across phases, including reasons for non-participation in Phase V voting, is presented in Supplemental Table S1 in Additional File 1.

### CVR: domain and subitem level validation

**Fig. 2:**
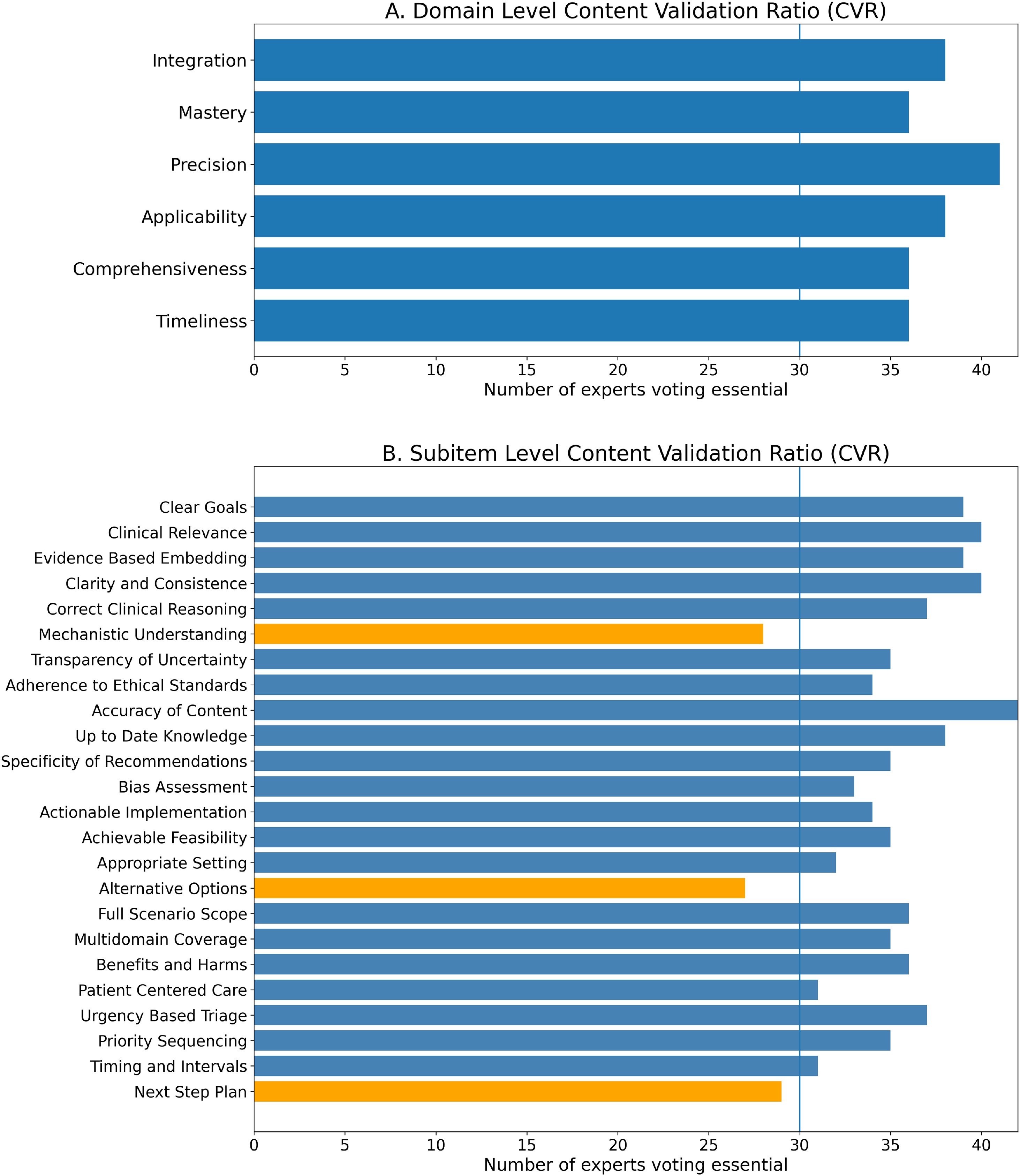
Content Validation Results for the IMPACT Framework. Panel consensus voting results showing the number of panelists rating each item as essential. (A) Domain level validation: all six domains exceeded the 70% agreement threshold (vertical line, n = 30). (B) Subitem level validation: blue bars indicate retained subitems; orange bars indicate three subitems removed due to insufficient consensus (Mechanistic Understanding, Alternative Options, and Next Step Plan).

The candidate IMPACT framework comprised six domains and 24 candidate subitems. Based on exact binomial probability for a panel of 42 panelists (α = 0.05), the minimum statistical threshold was CVR critical = 0.31, requiring at least 28 panelists to rate an item as necessary. We adopted a more stringent a priori criterion of 70% agreement, corresponding to CVR ≥ 0.40 (at least 30 panelists). All six domains exceeded this criterion, with Precision receiving the highest endorsement (Fig. 2A). The mean domain-level agreement was 89.3% (37.5/42 panelists, CVR = 0.79).

At the subitem level, 21 of the original 24 subitems met the retention criterion, with Accuracy of Content achieving unanimous endorsement (Fig. 2B). The mean agreement among retained subitems was 85.7% (36/42 panelists, CVR = 0.71). Three subitems failed to reach the 70% threshold: Mechanistic Understanding (66.7%), Alternative Options (64.3%), and Next-Step Planning (69.0%). After steering committee review, these were removed due to conceptual overlap with retained subitems. To address potential circularity, CVR was recalculated after removing the steering committee votes (N = 34). All six domains and all 21 retained subitems continued to exceed the threshold, and the same three subitems were removed (Supplemental Table S2 in Additional File 1).

### Content validity index: domain and subitem level validation

**Fig. 3:**
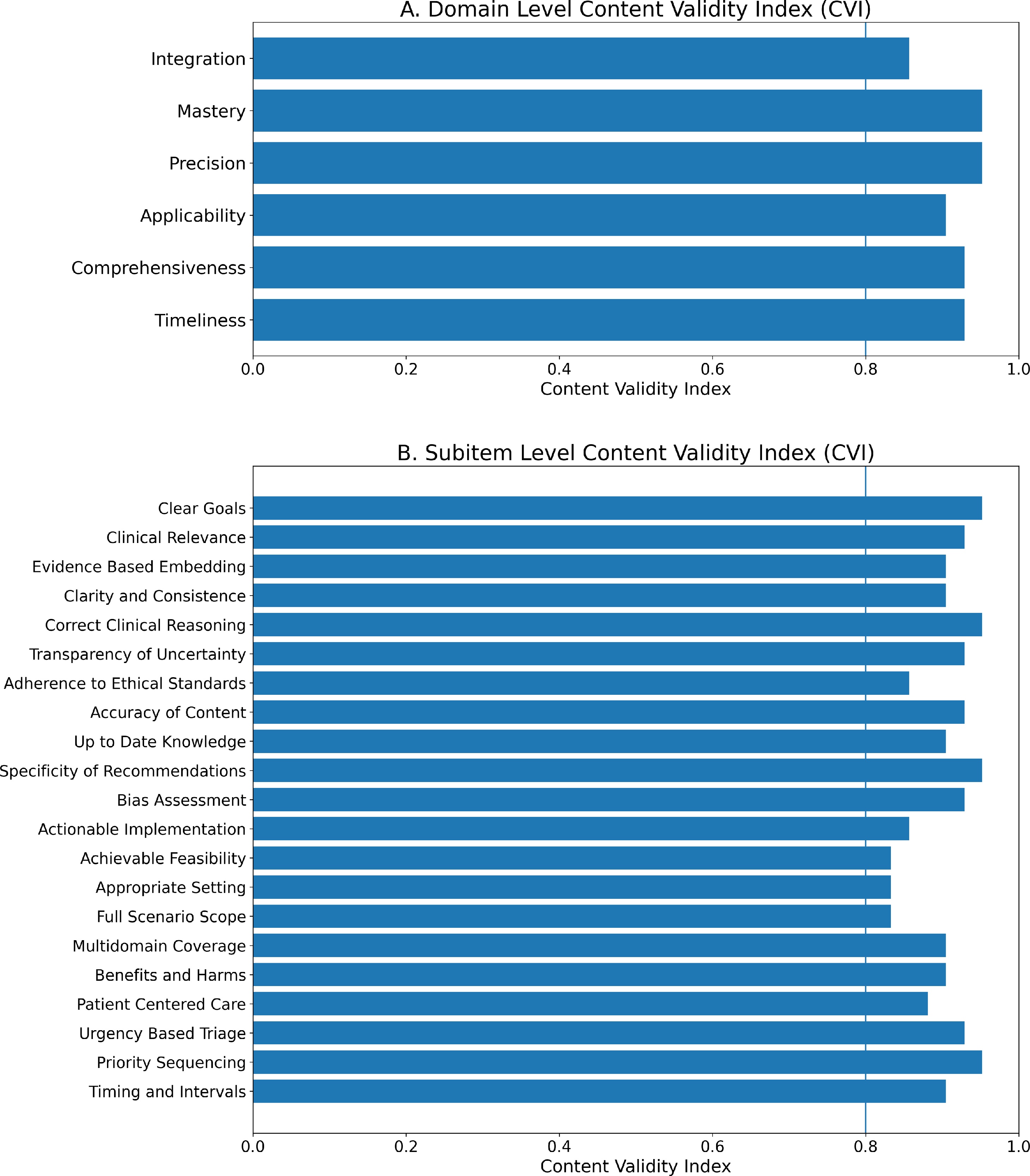
Item-Level Content Validity Index (I-CVI) for the IMPACT Framework. Content validity index results based on panelist ratings. (A) Domain level I-CVI: all six domains exceeded the 0.80 threshold (vertical line), with values ranging from 0.86 (Integration) to 0.95 (Mastery and Precision). (B) Subitem level I-CVI: all 21 retained subitems met the validity threshold (I-CVI ≥0.80), with Correct Clinical Reasoning, Specificity of Recommendations, and Priority Sequencing achieving the highest values.

All six domains exceeded the predefined I-CVI threshold of 0.80, with Mastery and Precision achieving the highest relevance and a mean I-CVI of 0.92 (Fig. 3A). All 21 retained subitems exceeded the I-CVI threshold, with a mean I-CVI of 0.90 (Fig. 3B). To address potential circularity, I-CVI was recalculated after removing the steering committee votes (N = 34). All six domains and 19 of 21 retained subitems continued to exceed the threshold of 0.80; two subitems scored 0.79 (Supplemental Table S3 in Additional File 1).

### Validated IMPACT framework

The final IMPACT framework comprises six domains and 21 subitems. Integration, Precision, and Comprehensiveness each contain four subitems, while Mastery, Applicability, and Timeliness each contain three subitems. Panel consensus definitions for each of the six IMPACT domains are presented in Table 1. Panel consensus descriptions for each of the 21 validated subitems are presented in Table 2.

**Table 1:** Panel consensus definitions of the six IMPACT domains.

Domains	Definition
Integration	Evaluates whether GenAI–derived clinical information coherently integrates clear clinical goals, patient-specific critical care context, and evidence-based knowledge into a clear, consistent, and clinically usable response
Mastery	Assesses whether GenAI demonstrates expert-level clinical reasoning, transparent acknowledgment of uncertainty, and consistent adherence to ethical standards in critical care decision-making
Precision	Verifies whether GenAI–derived clinical information is precise, accurate, up to date, specific, and unbiased, enabling reliable and clinically actionable recommendations in critical care
Applicability	Evaluates whether GenAI–derived clinical information provides actionable and feasible recommendations that are appropriate to the patient’s condition and the critical care setting
Comprehensiveness	Examines whether GenAI–derived clinical information provides comprehensive, multidomain coverage of the clinical scenario, addressing benefits, risks, and patient-centered decision-making considerations
Timeliness	Determines whether GenAI–derived clinical information appropriately prioritizes urgency, sequences interventions by risk, and incorporates context-sensitive timing and reassessment intervals

GenAI, Generative artificial intelligence.

**Table 2:** Panel consensus descriptions of the 21 validated IMPACT subitems.

Domains	Subitems	Description
Integration	Clear Goals	Evaluate whether GenAI explicitly identifies clear clinical goals or objectives of care
	Clinical Relevance	Assess if GenAI response is patient-specific, context-aware, and not merely generic
	Evidence-Based Embedding	Check if GenAI incorporates evidence-based knowledge, guidelines, and standard clinical protocols
	Clarity and Consistence	Appraise response quality for clarity, logical organization, and internal consistency
Mastery	Correct Clinical Reasoning	Assess pathophysiological reasoning, clinical priorities, and medically valid cause-and-effect conclusions
	Transparency of Uncertainty	Determine if GenAI acknowledges uncertainty, limitations, and knowledge gaps, avoiding false certainty
	Adherence to Ethical Standards	Verify that GenAI upholds patient autonomy, rights, safety, fairness, and professional conduct
Precision	Accuracy of Content	Verify whether the GenAI provides factually accurate information consistent with established clinical evidence
	Up-to-Date Knowledge	Check whether the GenAI uses current medical knowledge aligned with recent evidence and guidelines
	Specificity of Recommendations	Assess whether GenAI delivers patient-specific recommendations with actionable steps and clinical parameters
	Bias Assessment	Evaluate whether the GenAI identifies or avoids bias, unsupported assumptions, or imbalanced clinical perspectives
Applicability	Actionable Implementation	Determine whether GenAI provides clear, practical clinical steps that can be immediately implemented
	Achievable Feasibility	Assess whether recommendations are realistically achievable given patient condition and available resources
	Appropriate Setting	Evaluate whether recommendations match ICU monitoring level, care intensity, and operational context
Comprehensiveness	Full Scenario Scope	Determine if GenAI anticipates deterioration risks and addresses the full clinical scenario comprehensively
	Multidomain Coverage	Evaluate whether GenAI covers relevant physiological systems, diagnostics, and management domains appropriately
	Benefits and Harms	Review how GenAI balances therapeutic benefits with risks, complications, and potential patient harms
	Patient-Centered Care	Verify if GenAI incorporates patient values, goals of care, and shared decisions appropriately
Timeliness	Urgency-Based Triage	Assess whether GenAI recognizes clinical urgency and identifies conditions requiring immediate attention
	Priority Sequencing	Evaluate whether GenAI sequences actions by clinical importance, risk level, and therapeutic priority
	Timing and Intervals	Examine whether GenAI adjusts timing and reassessment intervals based on evolving patient physiology

GenAI, Generative artificial intelligence; ICU, intensive care unit.

## Discussion

The IMPACT framework offers a structured approach for evaluating GenAI outputs in critical care settings, addressing a gap in existing evaluation methods. Through consensus involving panelists from 12 countries and regions, we identified six domains and 21 subitems that reflect what experienced clinicians consider essential when assessing GenAI clinical decision support. All six domains achieved strong agreement, averaging 89.3%, and retained subitems averaged 85.7%. Mean I-CVI values of 0.92 for domains and 0.90 for subitems supported solid content validity.

Current benchmark evaluations of medical AI often rely on automated metrics that correlate poorly with human clinical judgment and fail to assess how AI integrates into clinical workflows [30]. Clinical evaluation focusing on factual accuracy, clinical utility, and safety offers a more meaningful assessment approach [31]. The framework is named IMPACT because clinical impact is its central goal. Each domain addresses a distinct aspect: Integration evaluates coherence with clinical goals, patient context, and evidence; Mastery assesses clinical reasoning, uncertainty, and ethical standards; Precision verifies factual accuracy and current knowledge; Applicability ensures recommendations are actionable and feasible in practice; Comprehensiveness examines the full clinical scenario including benefits, harms, and patient-centered care; and Timeliness addresses urgency recognition and priority sequencing [1,31,32]. Although some relatedness exists between domains, each serves a different purpose. Integration focuses on coherence of the response, while Applicability focuses on real-world implementation. Mastery assesses reasoning quality, while Comprehensiveness examines clinical coverage. The consensus results support these distinctions, as all six domains independently exceeded validity thresholds (mean agreement 89.3%, I-CVI = 0.92). Precision achieved the highest agreement, confirming that factual accuracy remains the cornerstone of trustworthy clinical AI. Framework refinement involved removing three subitems due to insufficient consensus and conceptual overlap. Mechanistic Understanding, while valuable for teaching, was considered less essential when clinicians need actionable recommendations, overlapping with Correct Clinical Reasoning and Accuracy of Content. Alternative Options overlapped with Multidomain Coverage and Benefits and Harms. Next-Step Planning overlapped with Timing and Intervals. These removals maintained focus and eliminated redundancy.

The IMPACT framework complements recent large-scale benchmarking efforts. MedHELM introduced a clinician-validated taxonomy spanning five healthcare categories and 37 benchmarks, enabling systematic comparison of frontier LLMs through automated metrics and an LLM-jury approach [33]. MedHELM excels at scalable model comparison across broad healthcare tasks and provides a public leaderboard for the research community. However, its open-ended evaluation relies on three axes (accuracy, completeness, and clarity). The authors acknowledge that instance-level rubrics, uncertainty quantification, and evaluation in augmentative settings with human experts remain unaddressed. IMPACT fills these gaps. Domains such as Mastery assess clinical reasoning and uncertainty transparency. Timeliness evaluates urgency-based triage and priority sequencing. These dimensions are absent from current benchmarking frameworks. Where MedHELM supports model selection, IMPACT supports quality assessment of individual outputs at the bedside. The two approaches may serve as complementary layers in a comprehensive evaluation strategy for medical AI.

Among these six domains, Comprehensiveness warrants further elaboration given its unique role in capturing the multidimensional nature of critical care decision-making. Comprehensiveness reflects the interdependence of physiological systems and the embedding of clinical care within a broader biopsychosocial context. Although current AI models often perform well in predicting single outcomes such as mortality or isolated organ failure, they frequently underrepresent concurrent effects on other organ systems and rarely consider how clinical decisions interact with the psychosocial environment [9]. A genuinely comprehensive AI would integrate multimodal data sources beyond conventional physiological and laboratory inputs, including patient values, preferences, contextual factors, ethical trade-offs, and resource limitations [34]. This broader scope enables AI to generate outputs that are more clinically meaningful and generalizable across diverse settings [9]. When gaps in comprehensiveness are identified, mitigation strategies should include engagement of multidisciplinary teams and incorporation of diverse data sources beyond traditional electronic health record data.

The 21 validated subitems translate broad domains into practical evaluation criteria for bedside use [35]. Accuracy of Content achieved unanimous agreement, confirming that factual correctness is non-negotiable for clinical AI. Clarity and Consistency and Clinical Relevance ranked second and third, reflecting that AI outputs must be easy to understand and directly applicable to the patient at hand. These three subitems represent what clinicians universally expect from any reliable consultation [36]. The lowest agreement occurred for Timing and Intervals, Patient-Centered Care, and Appropriate Setting. These subitems involve concepts that require interpretation across diverse healthcare systems and cultural contexts [37]. ICU environments vary widely, from high-resource academic centers to resource-limited settings. Future validation studies should consider linguistic and cultural adaptation to ensure consistent interpretation internationally.

The validated IMPACT framework establishes the foundation for several planned developments. Validity and reliability are sequential steps in framework development. The current study established content validity through multinational consensus. Recent evidence demonstrates that granular evaluation rubrics achieve higher inter-rater reliability than traditional scales [[38], [39], [40]]. Building on this principle, we developed five sub-indicators for each subitem with standardized scoring anchors ranging from very poor to excellent. An example of the scoring criteria is presented in Supplemental Table S4 in Additional File 1. Preliminary reliability testing by the NSECC group has informed this development. In a separate reliability study, eight clinicians and an automated evaluator (o3-mini) independently scored LLM-generated clinical reports for ICU cases from the MIMIC-IV database using this scoring system, yielding good inter-rater reliability among clinicians (ICC = 0.836, 95% CI 0.792–0.876) and high reliability between clinicians and the automated evaluator (ICC = 0.975, 95% CI 0.969–0.982) (Yeh YC, et al., manuscript submitted for publication). Further details are provided in Supplemental Table S5 in Additional File 1. This level of reliability compares favorably with published benchmarks for clinical AI evaluation instruments, including recent studies demonstrating ICC values of 0.818 for LLM-based clinical evaluation [38]. In a separate study, the IMPACT framework was used to evaluate LLM-generated clinical reports that incorporated machine learning risk scores and explainability features in an ICU setting [41]. Criterion-related validity against clinical outcomes represents the next phase. Clinical vignettes will be developed for each sub-indicator to guide consistent scoring, with examples drawn from representative ICU cases including sepsis, acute respiratory distress syndrome, and acute kidney injury. As disease-specific GenAI clinical decision support systems emerge, we envision condition-specific extensions such as IMPACT-Sepsis and IMPACT-ARDS [42].

This study represents an initial step toward systematic evaluation of GenAI in critical care. Our work benefited from participation of 58 multinational panelists, using CVR and I-CVI calculations to quantify agreement. Several limitations warrant consideration. First, language may have influenced how non-English-speaking panelists interpreted definitions and descriptions; future validation should incorporate formal translation and cultural adaptation. Second, participation from resource-limited settings remained limited, potentially affecting generalizability. Third, GenAI encompasses diverse models with varying architectures; additional work is needed to understand how specific models demonstrate distinct strengths when evaluated using the IMPACT framework. Fourth, the current study established content validity but did not link IMPACT scores to clinical outcomes. A reliable framework to identify inaccurate or harmful GenAI outputs is a prerequisite for outcome studies. Linking IMPACT scores to patient outcomes through prospective studies remains an important next step. Fifth, AI technology is evolving rapidly. Like the Surviving Sepsis Campaign and ACLS guidelines, the IMPACT framework will require periodic revision. Agent-based systems may require even more rigorous output evaluation. The six core domains remain relevant across AI architectures, but subitems and scoring criteria will need updates as technology and practice evolve. Sixth, AI adoption is context-dependent. The Applicability domain addresses this by evaluating whether recommendations are feasible and appropriate for the local care environment. The framework serves as a roadmap rather than fixed criteria, and setting-specific adaptations represent an important future direction. Seventh, a single voting round was used because items had undergone refinement through Phases I to IV. All participation was voluntary, and resources for international coordination were limited. Iterative rounds with expanded international participation will require additional resources and represent an important future direction. Looking forward, AI will permeate critical care practice and has immense potential to support clinical decision-making, alleviate clinical burden, and improve patient outcomes [43]. Understanding how patients and families perceive AI-assisted care represents an essential future direction, as their perspectives will shape responsible integration of these technologies [44]. Recent consensus recommendations have similarly called for establishing a social contract for AI in healthcare that includes patient and societal representatives [45].

In conclusion, the IMPACT Evaluation Framework offers a structured approach for assessing GenAI outputs in critical care. Through multinational panel consensus, we proposed six domains and 21 subitems capturing key dimensions of AI quality, from factual precision to patient-centered care. As GenAI enters intensive care practice, systematic evaluation may help ensure patient safety. We have applied the framework to evaluate LLM-generated clinical reports in an ICU setting, confirming its feasibility [41]. We hope this framework serves as a foundation for clinicians and researchers working toward safe integration of AI into critical care medicine.

## Author contributions

YCY, KCS, TF, LL, WM, HYC, and WCC wrote the first draft. All authors have contributed to the discussion and to the final manuscript's discussion, revision, and approval.

## Consent for publication

All authors consent to this publication.

## Human ethics and consent to participate declarations

Not applicable.

## Declaration of Generative AI and AI-assisted technologies in the writing process

During the preparation of this work, the authors used GPT-5.2 and Claude Opus 4.5 for English editing. After using these tools, the authors reviewed and edited the content as needed and take full responsibility for the content of the published article.

## Funding

This work was partially supported by a grant from National Taiwan University Hospital(NTUH 114-FY0002 to YCY). The funder had no role in the study design, data collection, analysis, interpretation of results, or preparation of the manuscript.

## Data availability

The data supporting the findings of this study are available from the corresponding author upon reasonable request.

## Clinical trial number

Not applicable.

## Declaration of competing interest

The authors declare no competing interests. No panelist reported conflicts of interest during the review or voting process. The steering committee had no financial or institutional relationships that could have influenced the framework content or consensus outcomes.
