# Supplementary material for: The IMPACT framework for evaluating generative AI in critical care: development and multinational consensus validation
Source: Ann Intensive Care. 2026 May 12;16:100078. doi: 10.1016/j.aicoj.2026.100078 (PMC13195341; doi:10.1016/j.aicoj.2026.100078)
Supplement: Supplementary file 1 [file mmc1.pdf]

The IMPACT framework for evaluating generative AI in critical care: development and multinational consensus validation

Additional File 1

Content

1. ACCORD Checklist
2. Supplemental Table 1. Panelist Information
3. Supplemental Table 2. Content Validity Ratio Before and After Removing Steering Committee Votes
4. Supplemental Table 3. Item-Level Content Validity Index Before and After Removing Steering Committee Votes
5. Supplemental Table 4. Example Scoring Criteria Using IMPACT Framework
6. Supplemental Table 5. Summary of the IMPACT Framework Reliability Study

ACCORD Checklist – Manuscript Compliance

Manuscript: *IMPACT Framework (01_Manuscript_20260309.docx)*
Guideline: ACCORD (Gattrell et al., PLoS Med, 2024)
Compliance: **27/35 Met** | **7/35 Partial** | **1/35 Not Addressed**

Item #	Section	Item Wording	Manuscript Memo (Page #)
**TITLE (n = 1)**			
**T1**	Title	Identify the article as reporting a consensus exercise and state the consensus methods used in the title.	Met (p. 1). Title: "...Development and Multinational Consensus Validation." Consensus is identified. Method ("structured, multi-phase consensus methodology") detailed in Methods (p. 6).
**INTRODUCTION (n = 3)**			
**I1**	Introduction	Explain why a consensus exercise was chosen over other approaches.	Met (p. 5). "We used consensus methodology because no gold standard criteria exist for evaluating GenAI outputs in critical care, and empirical validation alone cannot capture the tacit clinical knowledge needed."
**I2**	Introduction	State the aim, intended audience, and geographical scope.	Met (p. 5). "Our aim is to provide a structured tool for evaluating GenAI clinical decision support in critical care." Audience: ICU clinicians, physician scientists, IT specialists. Scope: multinational, 12 countries.
**I3**	Introduction	If an update of existing document, state why and cite original.	Met (N/A). New framework, not an update of an existing document.
**METHODS – Registration (n = 1)**			
**M1**	Registration	If registered, state platform and link. If not, state so.	Partial (p. 6). "In the absence of a dedicated registry for panel consensus studies, we ensured methodological transparency by providing a comprehensive protocol description in the Methods.
**METHODS – Selection of SC and/or Panelists (n = 4)**			
**M2**	Selection of SC/Panelists	Describe the role(s) and expertise of those directing the consensus exercise.	Met (p. 6). Steering committee of 8 person listed (chair Y.C.Y., 7 members G.S.M., K.C.S., L.L., W.M., H.Y.C., W.C.C., M.C.S.). "Provided clinical and methodological oversight across all phases." M.C.S. identified as physician biostatistician.
**M3**	Selection of SC/Panelists	Explain criteria for panelist inclusion and rationale for numbers. State who selected.	Met (pp. 6–7). Eligibility: leadership in critical care societies, ICU practice, AI research expertise, or IT experience. Chair invited 60 panelists; nominations reviewed by chair and SC.
**M4**	Selection of SC/Panelists	Describe the recruitment process.	Met (pp. 6–7). "The chair personally invited 60 potential panelists via email, WhatsApp, and LINE, with reminder messages." Panelists could nominate others with SC approval.
**M5**	Selection of SC/Panelists	Describe the role of any public, patients, or carers.	Met (p. 7). "No patients, carers, or members of the public participated… as the framework was intended to evaluate GenAI clinical decision support content for clinicians in critical care."
**METHODS – Preparatory Research (n = 3)**			
**M6**	Preparatory Research	Describe how information was obtained prior to generating items.	Met (p. 7). Phase I: focused review of DISCERN, QUEST, HONcode, JAMA

Item #	Section	Item Wording	Manuscript Memo (Page #)
			benchmarks, DARTS. Candidate domains/subitems drafted from these foundations.
**M7**	Preparatory Research	Describe any systematic literature search in detail (strategy, dates).	Partial (p. 7). A "focused review" of 5 established frameworks was conducted.
**M8**	Preparatory Research	Describe how evidence was summarized and if provided to panelists.	Partial (p. 8). Phase IV: "Panelists received the draft materials, reviewed the contents, and provided feedback."
**METHODS – Assessing Consensus (n = 11)**			
**M9**	Assessing Consensus	Describe the methods and steps taken to reach consensus.	Met (pp. 6–8). Five-phase process described with Figure 1. "Structured, multi-phase consensus methodology": NSECC conceptualization, TSECCM adoption, symposium, panel review, and voting.
**M10**	Assessing Consensus	Describe how questions were presented and response options.	Met (pp. 8–9). Each domain/subitem presented as separate statement with definition. CVR: 3-point scale (1=necessary, 2=useful but not necessary, 3=not necessary). I-CVI: 4-point scale (1–4). Slido and Google Forms used.
**M11**	Assessing Consensus	State the objective of each consensus step.	Met (pp. 7–8). Phase I = concept initiation; Phase II = formal adoption; Phase III = interdisciplinary discussion; Phase IV = international panel review; Phase V = content validity through structured voting.
**M12**	Assessing Consensus	State the definition of consensus and rationale.	Met (p. 9). CVR ≥ 0.429 (70% necessary ratings), exceeding minimum critical CVR 0.29 for 42 panelists. I-CVI ≥ 0.80. "Higher than commonly used standards… clinical safety implications."
**M13**	Assessing Consensus	State whether items meeting consensus were included in subsequent voting rounds.	Met (p. 8). "A single voting round was conducted." No subsequent rounds; not applicable in the iterative sense.
**M14**	Assessing Consensus	Describe how responses were collected (group vs. individual).	Met (p. 8). "Voting was conducted during two online meetings using Slido for live polling, with Google Forms available." Group online meetings with individual anonymous voting.
**M15**	Assessing Consensus	Describe how responses were processed/synthesized.	Met (pp. 9–10). CVR and I-CVI formulas described in detail. Conservative assignment for missing ratings. Data Handling section describes aggregated analysis.
**M16**	Assessing Consensus	Describe any piloting of study materials/survey instruments.	Partial (p. 7). Phase III symposium served as a pilot.
**M17**	Assessing Consensus	Describe how feedback was provided to panelists at each step.	Partial (p. 7-8). Describe feedbacks during different phase.
**M18**	Assessing Consensus	State whether anonymity was planned and how maintained.	Met (p. 10). "Voting was anonymous at the panel level; panelists could not see how others voted. The chair maintained a participant roster… Only the chair could link participants to responses."
**M19**	Assessing Consensus	State if the SC was involved in decisions made by the consensus panel.	Met (p. 10). "Chair and steering committee members participated in Phase V voting, and their votes were included in CVR and I-CVI calculations." SC also approved wording revisions and supervised comment synthesis.
**METHODS – Participation (n = 2)**			
**M20**	Participation	Describe any incentives used to encourage	Met (p. 7). "Participation was voluntary

Item #	Section	Item Wording	Manuscript Memo (Page #)
		participation.	without financial incentives or reimbursement.” Reminder messages via email, WhatsApp, LINE mentioned.
**M21**	Participation	Describe any adaptations for accessibility.	Met (p. 8). “Meetings and survey materials were primarily in English, with a Mandarin instruction document provided for Taiwanese panelists.” Google Forms backup for technical issues.
**RESULTS (n = 5)**			
**R1**	Results	State when the exercise was conducted, dates and timeline.	Met (pp. 7–8, Figure 1). Phase I (Nov 2024), Phase II (Jan 2025), Phase III (Oct 2025), Phase IV (Nov 2025), Phase V (Dec 2025). Full timeline provided.
**R2**	Results	Explain any deviations from the study protocol.	Partial (p. 6). No formal pre-registered protocol to deviate from.
**R3**	Results	Report quantitative and qualitative data on participating panelists for each step.	Met (p. 10). 58 panelists in review, 42 in voting. Breakdown by profession (34 intensivists, 5 non-ICU physicians, 5 IT faculty, 4 IT engineers, 3 nurses, 2 pharmacists, etc.) and by 12 countries with numbers.
**R4**	Results	Report the final outcome as qualitative and/or quantitative data.	Met (pp. 10–11, Figures 2–3, Tables 1–2). CVR and I-CVI for all domains (mean 89.3%, CVR=0.79, I-CVI=0.92) and subitems (mean 85.7%, CVR=0.71, I-CVI=0.90).
**R5**	Results	List items modified or removed, with reasons and timing.	Met (pp. 11, 12–13). Three subitems removed: Mechanistic Understanding (66.7%), Alternative Options (64.3%), Next-Step Planning (69.0%). Reasons: insufficient consensus and conceptual overlap. Discussion elaborates overlap rationale.
**DISCUSSION (n = 2)**			
**D1**	Discussion	Discuss methodological strengths and limitations.	Met (pp. 14–15). Strengths: 58 multinational panelists, CVR and I-CVI. Limitations: language influence, limited resource-limited settings, diverse GenAI architectures, single voting round.
**D2**	Discussion	Discuss consistency with preexisting literature.	Met (pp. 12–14). Compares with automated metrics, DISCERN, existing frameworks (TRIPOD+AI, DECIDE-AI, CHART). Explains why IMPACT addresses gaps not covered by current tools.
**OTHER INFORMATION (n = 3)**			
**O1**	Other	List endorsing organizations and their role.	Met (pp. 7–8). TSECCM (formal task, Phase II), TSCCM and TSA (joint symposium, Phase III). Roles described in development timeline.
**O2**	Other	State COI among SC and panelists; describe how managed.	Partial (p. 17). “The authors declare no competing interests.”
**O3**	Other	State funding received and the role of the funder.	Partial (p. 17). “Partially supported by a grant from NTUH (NTUH 114-FY0002 to YCY).” Funder’s role in study design/execution not explicitly described.

Supplemental Table 1. Panelist Information

Name	Country	Expertise	Phase IV	Phase V
**International Members**			**22**	**17**
Abdul Jabbar Bin Ismail	Malaysia	Intensivist	V	V
Ahsina Jahan	Bangladesh	Intensivist	V	V
Bernard Cholley	France	Intensivist	V	
David Ku	Australia	Intensivist	V	V
David Pilcher	Australia	Intensivist, Medical Informatics	V	V
Daniel De Backer	Belgium	Intensivist	V	
Fong Kean Khang	Malaysia	Intensivist	V	V
Greg S. Martin	USA	Intensivist	V	V
Ho-Geol Ryu	South Korea	Intensivist	V	
Leo Celi	USA	Intensivist, Medical Informatics	V	
Lowell Ling	Hong Kong	Intensivist	V	V
Mengling Feng	Singapore	Information Technology	V	
Moritoki Egi	Japan	Intensivist	V	V
Natachai Srisawat	Thailand	Nephrologist	V	
Rishikesan Kamaleswaran	USA	Information Technology	V	
Kay Choong SEE	Singapore	Intensivist, Medical Informatics	V	V
Sungwon Na	South Korea	Intensivist, Medical Informatics	V	V
Sin-Chee Tan	Australia	Intensivist, Medical Informatics	V	V
Tina Chen	USA	Intensivist	V	
Tomoko Fujii	Japan	Intensivist	V	V
Wasinee nart Mongkolpun	Thailand	Intensivist	V	V
Wing-Sum Chan	Hong Kong	Anesthesiologist, Medical Informatics	V	V
**Taiwan Members**			**36**	**25**
Chao-Chun Chuang	Taiwan	Information Technology	V	V
Chi-Ju Yang	Taiwan	Pharmacist	V	V
Chien-Chang Lee	Taiwan	Emergency Physician, Medical Informatics	V	
Chien-Hao Chen	Taiwan	Pharmacist	V	
Chien-Kun Ting	Taiwan	Anesthesiologist, Medical Informatics	V	
Ching-Tang Chiu	Taiwan	Intensivist	V	
Ching-Yuan Chan	Taiwan	Critical Care Nurse	V	
Chueng-He Lu	Taiwan	Intensivist, Anesthesiologist	V	
Daniel Fu-Chang Tsai	Taiwan	Family Physician, Ethicist	V	
Fei-Pi Lai	Taiwan	Information Technology	V	
Hou-Tai Chang	Taiwan	Intensivist	V	V
Hsiang-Wei Hu	Taiwan	Information Technology	V	V
Hsiao-Lan Shih	Taiwan	Nurse	V	
Hsuan-Yu Chen	Taiwan	Orthopedic Surgeon Medical Informatics	V	
Kuan-Fu Chen	Taiwan	Emergency Physician, Medical Informatics	V	
Kuan-Yu Chen	Taiwan	Information Technology	V	
Kuang-Hua Cheng	Taiwan	Intensivist	V	
Kuo-Ching Yuan	Taiwan	Intensivist	V	V
Lok-Hi Chow	Taiwan	Intensivist, Anesthesiologist	V	
Min-Shan Tsai	Taiwan	Intensivist, Emergency Physician	V	
Ming-Cheng Chan	Taiwan	Intensivist	V	
Ming-Chieh Shih	Taiwan	Physician biostatistician	V	V
Pei-Fu Chen	Taiwan	Anesthesiologist, Medical Informatics	V	V
Po-Chih Kuo	Taiwan	Information Technology	V	V
Po-Hao Hsu	Taiwan	Information Technology	V	
Sheng-Ru Lai	Taiwan	Dietitian	V	
Shih-Chi Ku	Taiwan	Intensivist	V	V
Wei-Cheng Chen	Taiwan	Intensivist, Medical Informatics	V	V
Wei-Ling Hsiao	Taiwan	Critical Care Nurse	V	V
Wei-Chung Wang	Taiwan	Information Technology	V	
Ya-Wen Yang	Taiwan	Intensivist, Surgeon	V	V
Young-Jen Lin	Taiwan	Intensivist, Surgeon	V	V
Yih-Sharng Chen	Taiwan	Surgeon, ECMO expert	V	V
Yu-Chang Yeh	Taiwan	Intensivist, Medical Informatics	V	V
Yu-Chen Chuang	Taiwan	Information Technology	V	V
Yin-Yi Han	Taiwan	Intensivist	V	V

Supplemental Table 2. Content Validity Ratio Before and After Removing Steering Committee Votes

	Full panel (N = 42)		Sensitivity analysis (N = 34)		
**Domain / Subitem**	**Ne**	**CVR**	**Ne**	**CVR**	**Status**
**Integration**	38	0.81	30	0.76	—
**Mastery**	36	0.71	29	0.71	—
**Precision**	41	0.95	33	0.94	—
**Applicability**	38	0.81	30	0.76	—
**Comprehensiveness**	36	0.71	28	0.65	—
**Timeliness**	36	0.71	29	0.71	—
**Subitems**					
Clear Goals	39	0.86	31	0.82	Retained
Clinical Relevance	40	0.90	32	0.88	Retained
Evidence-Based Embedding	39	0.86	32	0.88	Retained
Clarity and Consistency	40	0.90	32	0.88	Retained
Correct Clinical Reasoning	37	0.76	30	0.76	Retained
Mechanistic Understanding	28	0.33	22	0.29	Removed
Transparency of Uncertainty	35	0.67	28	0.65	Retained
Adherence to Ethical Standards	34	0.62	28	0.65	Retained
Accuracy of Content	42	1.00	34	1.00	Retained
Up-to-Date Knowledge	38	0.81	30	0.76	Retained
Specificity of Recommendations	35	0.67	28	0.65	Retained
Bias Assessment	33	0.57	26	0.53	Retained
Actionable Implementation	34	0.62	26	0.53	Retained
Achievable Feasibility	35	0.67	27	0.59	Retained
Appropriate Setting	32	0.52	25	0.47	Retained
Alternative Options	27	0.29	21	0.24	Removed
Full Scenario Scope	36	0.71	28	0.65	Retained
Multidomain Coverage	35	0.67	27	0.59	Retained
Benefits and Harms	36	0.71	29	0.71	Retained
Patient-Centered Care	31	0.48	24	0.41	Retained
Urgency-Based Triage	37	0.76	29	0.71	Retained
Priority Sequencing	35	0.67	27	0.59	Retained
Timing and Intervals	31	0.48	24	0.41	Retained
Next-Step Planning	29	0.38	22	0.29	Removed

Note: Sensitivity analysis performed by excluding eight steering committee members' votes. Full panel N = 42; sensitivity analysis N = 34. CVR = (Ne − N/2) / (N/2). The retention threshold was set at 70% agreement (CVR ≥ 0.40 for N = 42; CVR ≥ 0.41 for N = 34). All retained domains and subitems exceeded the threshold in both analyses. The same three subitems were removed in both analyses.

Abbreviations: CVR, Content Validity Ratio; Ne, number of panelists rating the item as necessary.

Supplemental Table 3. Item-Level Content Validity Index Before and After

Removing Steering Committee Votes

Domain / Subitem	Full panel (N = 42)		Sensitivity analysis (N = 34)		Status
	Ne	I-CVI	Ne	I-CVI	
**Integration**	36	0.86	29	0.85	—
**Mastery**	40	0.95	32	0.94	—
**Precision**	40	0.95	32	0.94	—
**Applicability**	38	0.90	30	0.88	—
**Comprehensiveness**	39	0.93	31	0.91	—
**Timeliness**	39	0.93	31	0.91	—
**Retained subitems**					
Clear Goals	40	0.95	32	0.94	Retained
Clinical Relevance	39	0.93	31	0.91	Retained
Evidence-Based Embedding	38	0.90	30	0.88	Retained
Clarity and Consistency	38	0.90	30	0.88	Retained
Correct Clinical Reasoning	40	0.95	32	0.94	Retained
Transparency of Uncertainty	39	0.93	31	0.91	Retained
Adherence to Ethical Standards	36	0.86	29	0.85	Retained
Accuracy of Content	39	0.93	32	0.94	Retained
Up-to-Date Knowledge	38	0.90	30	0.88	Retained
Specificity of Recommendations	40	0.95	32	0.94	Retained
Bias Assessment	39	0.93	31	0.91	Retained
Actionable Implementation	36	0.86	28	0.82	Retained
Achievable Feasibility	35	0.83	27	0.79	Marginal < 0.8
Appropriate Setting	35	0.83	28	0.82	Retained
Full Scenario Scope	35	0.83	27	0.79	Marginal < 0.8
Multidomain Coverage	38	0.90	30	0.88	Retained
Benefits and Harms	38	0.90	30	0.88	Retained
Patient-Centered Care	37	0.88	29	0.85	Retained
Urgency-Based Triage	39	0.93	31	0.91	Retained
Priority Sequencing	40	0.95	32	0.94	Retained
Timing and Intervals	38	0.90	30	0.88	Retained

Note: Sensitivity analysis performed by excluding eight steering committee members' votes. Full panel N = 42; sensitivity analysis N = 34. I-CVI = proportion of panelists rating the item as relevant (score 3 or 4 on a 4-point scale). The predefined acceptance criterion was I-CVI ≥ 0.80. All six domains exceeded the threshold in both analyses. Among the 21 retained subitems, Achievable Feasibility (I-CVI = 0.79) and Full Scenario Scope (I-CVI = 0.79) were marginally below the threshold in the sensitivity analysis but met the criterion in the full panel analysis.

Abbreviations: I-CVI, Item-level Content Validity Index; Ne, number of panelists rating the item as relevant.

Supplemental Table 4. Example Scoring Criteria Using IMPACT Framework

Item	Score	Description
Part 1: Evaluation of the Interpretation of Risk Factors		
1.1 Integration – Clinical Relevance	0	No relevant risk factors mentioned. *(Example: Omitting key factors such as intubation status or vital signs)*
	1	Bare mention of risk factors without context or proper interpretation. *(Example: Simply listing "intubation" without linking it to the patient's risk outcome)*
	2	Basic identification of risk factors without explicit reference to SHAP values, or includes misinterpretation. *(Example: Listing intubation and SpO_2 as risk factors without showing their SHAP values or clarifying their true clinical impact)*
	3	Clear explanation that includes numerical SHAP values to identify risk factors. *(Example: Explains that intubation increases risk by citing its SHAP value, but with limited integration of broader clinical context)*
	4	Detailed integration of numerical SHAP values with thorough clinical correlation and context, accurately relating both risk-enhancing and protective factors. *(Example: Thoroughly explains how the high SHAP value for intubation and the low or negative SHAP values for normal variables combine to define the patient's overall risk profile)*
1.2 Integration – Clarity of Information	0	The interpretation is disorganized and unclear, lacking a coherent structure or logical flow.
	1	The interpretation is partially clear with noticeable ambiguities that impede full understanding of the risk factors.
	2	The interpretation is generally clear but only provides a basic structure without exploring potential causes of the risk factors.
	3	The interpretation is clear and organized, with a logical flow and mention of at least one potential cause of the risk factors.
	4	The interpretation is exceptionally clear and well-organized, seamlessly presenting multiple distinct potential causes of the risk factors.
1.3 Mastery – Balance and Bias Assessment	0	One-sided – Only risk-enhancing factors are discussed, with no mention of protective factors.
	1	Minimal balance – Protective factors are mentioned but are mischaracterized or insufficiently addressed.
	2	Moderately balanced – Both risk-enhancing and protective factors are mentioned, but the protective factors receive limited or imprecise emphasis.
	3	Balanced – Both risk-enhancing and protective factors are clearly discussed, with a mild emphasis on protective factors and overall fair balance.
	4	Well-balanced – A comprehensive and thorough discussion of both risk-enhancing and protective factors is provided, with detailed and accurate emphasis on the protective factors.
1.4 Mastery – Uncertainty Acknowledgment	0	No acknowledgment of uncertainty, limitations, or assumptions is provided when they are clearly relevant.
	1	Minimal acknowledgment of uncertainty is present but lacks detail and does not explore how risk factors might interact.
	2	A basic acknowledgment of uncertainty is made with a brief mention of limitations or assumptions, or in cases where the predicted risk is extremely low (<2% ICU mortality) the absence of detailed uncertainty discussion is acceptable.
	3	A clear acknowledgment of uncertainty is provided, detailing potential interactions among risk factors and limitations in the interpretation.
	4	A comprehensive acknowledgment of uncertainties is offered, with an in-depth discussion of assumptions, limitations, and potential for further deterioration.
1.5 Precision – Accuracy of Content	0	The interpretation is incorrect, with major misinterpretations of key risk factors and their impact.
	1	The interpretation is mostly inaccurate, containing notable errors or misrepresentations of the clinical significance of the risk factors.
	2	The interpretation is partially accurate, correctly identifying some risk factors but overemphasizing minor details or missing the overall clinical context.

	3	The interpretation is mostly accurate, adequately reflecting the clinical severity of the risk factors with proper integration of the SHAP values.
	4	The interpretation is fully accurate, thoroughly incorporating numerical SHAP values to clearly and precisely reflect the clinical severity and implications of each risk factor.
1.6 Precision – Knowledge Depth and Currency	0	Superficial – The response lacks depth and fails to integrate current clinical evidence or detailed reasoning.
	1	Limited – The response offers a basic, student-level interpretation with minimal integration of current clinical insights.
	2	Adequate – The response demonstrates acceptable depth and understanding typical of a junior resident, though some nuances may be missing.
	3	Good – The response shows in-depth reasoning with current clinical insight appropriate for a senior resident, clearly explaining key risk factors and their significance.
	4	Expert-level – The response exhibits advanced, nuanced clinical understanding with comprehensive integration of the latest evidence, characteristic of an attending physician.
1.7 Applicability – Patient-specific Considerations	0	Superficial – The response ignores key patient-specific factors and provides a generic, non-tailored analysis.
	1	Limited – The response mentions patient-specific details in a superficial manner without adequate explanation of their significance.
	2	Adequate – The response identifies most risk factors but lacks depth in explaining how these factors interact specifically in the patient's context.
	3	Good – The response clearly explains the major risk factors and their clinical impact with appropriate patient-specific considerations, though without extensive nuance.
	4	Expert-level – The response provides a comprehensive and nuanced analysis that thoroughly integrates each patient-specific factor and their interactions, offering detailed clinical rationale.
1.8 Comprehensiveness – Complete Scope	0	Major omissions – The response omits many key risk factors, providing an incomplete picture.
	1	Several missing – Multiple important risk factors are not addressed, resulting in a noticeably incomplete analysis.
	2	Mostly complete – Most of the major risk factors are mentioned, but some relevant details or interactions are missing.
	3	Comprehensive – Nearly all critical risk factors and their interactions are addressed, though with minor omissions in detail.
	4	Exhaustive – Every relevant risk factor is clearly detailed, including nuanced interplay and critical conditions.
1.9 Timeliness – Response Urgency	0	No appropriate urgency – The response fails to match the risk profile, neither identifying critical factors in high-risk cases nor reassuring stability in low-risk scenarios.
	1	Minimal urgency – The response mentions risk factors but underemphasizes life-threatening conditions in high-risk cases or overstates them in stable situations.
	2	Moderate urgency – The response recognizes risk factors, identifying potential life-threatening conditions in high-risk cases or confirming stability in low-risk scenarios.
	3	High urgency – The response distinguishes key risk factors by prioritizing recognition of critical conditions in high-risk cases or confidently confirming stability in low-risk situations.
	4	Critical calibration – The response flawlessly aligns with the risk profile by precisely identifying critical conditions in high-risk cases or accurately assuring stability in low-risk scenarios.

Note: Each sub-indicator is scored 0–4. Subitem scores are converted to a 0–100 scale by multiplying by 25. This table presents Part 1 (Evaluation of the Interpretation of Risk Factors) as an example. The complete scoring criteria are described in the accepted feasibility study [41].
Abbreviations: SHAP, SHapley Additive exPlanations; SpO_2, peripheral oxygen saturation.

Supplemental Table 5. Summary of the IMPACT Framework Reliability Study

Item	Description
Study design	Retrospective offline evaluation
Database	MIMIC-IV v3.1, split into development, validation, and test cohorts by ICU admission year
Study Population	Development cohort: 30 cases<br>Validation cohort: 1,200 cases<br>Test cohort: 4,000 cases
Evaluation framework	IMPACT framework (25 items, 0–100 scale)
GenAI models evaluated	GPT-4o and GPT-5-mini
Automated evaluator	o3-mini
Clinical raters	Eight clinicians
Machine learning model	XGBoost for ICU mortality prediction with SHAP explainability
Prompting strategies	**P1:** Structured single-turn<br>**P2:** Task decomposition<br>**P3:** Multi-turn sequential
Primary outcome	Inter-rater reliability of IMPACT scores (ICC)
ICC among clinical raters	0.836 (95% CI 0.792–0.876)
ICC between clinical raters and automated evaluator	0.975 (95% CI 0.969–0.982)
GPT-4o test cohort scores	P1: 68.8 (3.0); P2: 75.9 (3.3); P3: 90.4 (4.1) P1 vs P3 and P2 vs P3: both $P < 0.001$
GPT-5-mini test cohort scores	Higher than GPT-4o across all strategies; P3: 98.2 (1.4)
Conclusion	The IMPACT framework demonstrated feasible and reliable evaluation of GenAI-generated clinical support information. Multi-turn prompting improved quality scores.
Reference	Yeh YC, et al., manuscript submitted for publication.

Abbreviations: CI, confidence interval; GenAI, generative artificial intelligence; ICC, intraclass correlation coefficient; ICU, intensive care unit; IMPACT, Integration, Mastery, Precision, Applicability, Comprehensiveness, and Timeliness; MIMIC, Medical Information Mart for Intensive Care; SHAP, SHapley Additive exPlanations.
